# A Case Report of Graves' Disease Induced by the Anti-Human Programmed Cell Death-1 Monoclonal Antibody Pembrolizumab in a Bladder Cancer Patient

**DOI:** 10.1155/2019/2314032

**Published:** 2019-10-17

**Authors:** Ken Yajima, Yushi Akise

**Affiliations:** ^1^Division of Diabetes, Endocrinology and Metabolism, Department of Internal Medicine, Federation of National Public Service Personnel Mutual Aid Associations Tachikawa Hospital, 4-2-22 Nishikicho, Tachikawa, Tokyo 190-8531, Japan; ^2^Department of Urology, Federation of National Public Service Personnel Mutual Aid Associations Tachikawa Hospital, 4-2-22 Nishikicho, Tachikawa, Tokyo 190-8531, Japan

## Abstract

Immune checkpoint inhibitors, such as anti-programmed cell death-1 (anti-PD-1), have been widely used in the treatment of malignancies. However, these drugs can cause immune-related adverse events resembling autoimmune diseases. There are some reports of Graves' disease (GD) induced by anti-cytotoxic T-lymphocyte-associated antigen 4 antibodies, but reports which discussed GD induced by anti-PD-1 antibodies are very rare. We report the case of a 61-year-old man with bladder cancer who presented with severe diarrhea, fatigue, palpitation, body weight loss, and hyperthyroidism after the fifth treatment with the anti-PD-1 monoclonal antibody pembrolizumab. His thyroid function prior to pembrolizumab administration had been subclinical hyperthyroidism, despite a negative thyroid-stimulating hormone receptor antibody (TRAb) level. On admission, pembrolizumab administration was discontinued. Graves' disease was diagnosed based on a positive TRAb test result and the ultrasonographic finding of increased blood flow in the superior thyroid artery. Based on colonoscopy findings, the cause of diarrhea was diagnosed as active colitis. His diarrhea was improved with prednisolone, and thyroid function was treated with potassium iodide and thiamazole. This case report of GD with positive TRAb induced by the anti-PD-1 antibody pembrolizumab may contribute to the understanding of the mechanism underlying the association between GD and autoimmune activation via PD-1.

## 1. Introduction

Immune checkpoint inhibitors (ICIs), including anti-programmed cell death-1 (PD-1), anti-programmed cell death-ligand 1 (PD-L1), and anti-cytotoxic T-lymphocyte-associated antigen-4 (CTLA4) monoclonal antibodies, are promising novel agents for advanced malignancies in recent years. PD-1 expressed on T cells and its ligands PD-L1 inhibit T-cell proliferation and cytokine production in activated T lymphocytes. CTLA4 is also expressed on T cells and exerts a suppressive effect on the immune response after the interaction between T-cells and antigen-presenting cells. These ICIs upregulate antitumor immune responses by blocking PD-1 and CTLA4 pathways. However, these drugs are associated with immune-related adverse events (irAEs) involving multiple endocrinology organs. Most thyroid dysfunction irAEs are destructive thyroiditis and hypothyroidism [1]. Graves' disease (GD) as an irAE is very rare; there are only a few reports of GD induced by anti-CTLA4 antibodies [2, 3]. However, to our knowledge, to date there are even fewer reports of GD caused by anti-PD-1 or anti-PD-L1 antibodies. We herein report a case of GD presenting with severe diarrhea in a patient with bladder cancer who was receiving the anti-PD-1 antibody pembrolizumab.

## 2. Case Presentation

A man aged 61 years was diagnosed with bladder cancer, with the primary lesion invading the prostate; he underwent total cystectomy, urethral resection, and ileal conduit two months later. After five months, computed tomography and magnetic resonance imaging showed retroperitoneal dissemination and para-aortic lymph node metastasis. Although he was treated with gemcitabine and carboplatin, he relapsed three months later. He was referred to our hospital to begin treatment with the anti-human PD-1 monoclonal antibody pembrolizumab.

**Figure 1:**
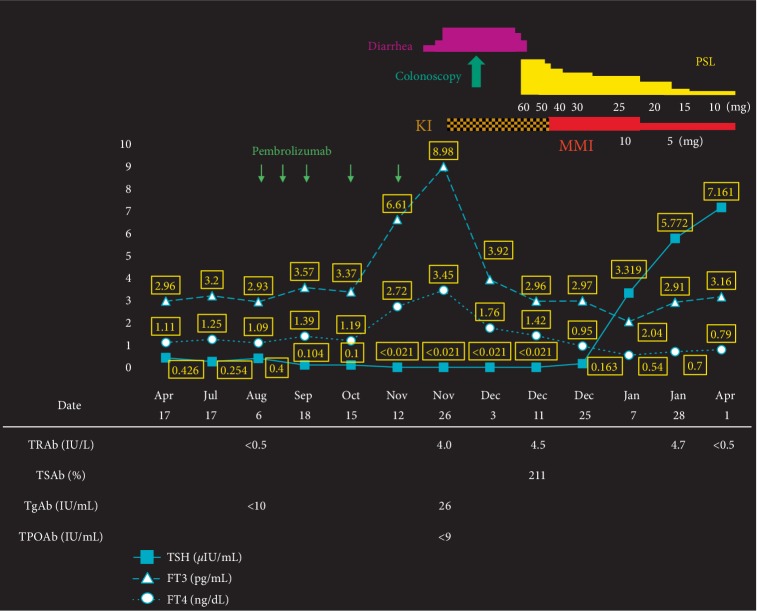
The clinical course including the patient's symptoms, medication administered, data of thyroid function tests and thyroid antibodies. The dates of pembrolizumab administration are as follows: first administration, Aug 7, 2018; second, Aug 27, 2018; third, Sep 18, 2018; fourth, Oct 15, 2018; fifth, Nov 12, 2018. Abbreviations: FT3, free triiodothyronine; FT4, free thyroxine; KI, potassium iodide; MMI, methimazole; PSL, prednisolone; TgAb, thyroglobulin antibody; TPOAb, thyroid peroxidase antibody; TRAb, thyroid stimulating hormone receptor antibody; TSAb, thyroid-stimulating antibody; TSH, thyroid stimulating hormone.

**Figure 2:**
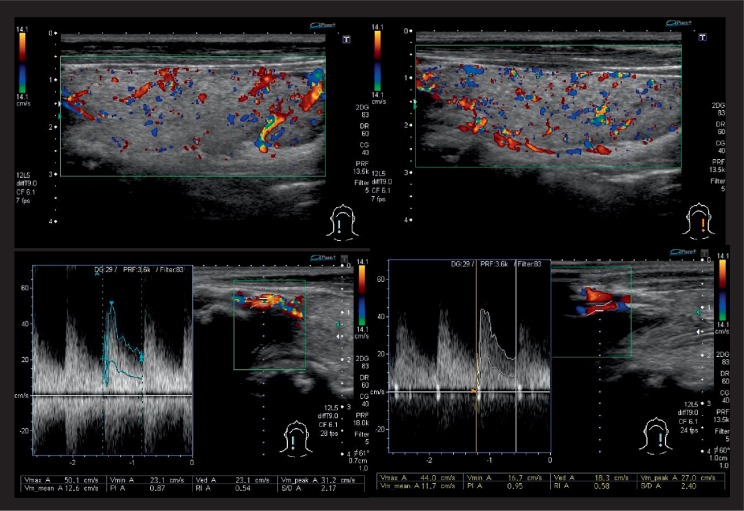
Thyroid ultrasonography performed the day after iodine treatment. Upper: Left is the intrathyroidal blood flow signal of the right lobe, Right is that of the left lobe. Lower: Left is right superior thyroid artery flow velocity, Vmax is 50.1 cm/s, Right is left superior thyroid artery flow velocity, Vmax is 44.0 cm/s.

Pembrolizumab (200 mg) was administered every three weeks, and it was effective. Five days after the fifth pembrolizumab administration (102 days after the first administration), he had two or three bouts of diarrhea per day. His symptoms gradually worsened; he was admitted to our hospital presenting with diarrhea 10×/day, fatigue, palpitation, and body weight loss. His blood pressure was 117/72 mmHg, body temperature was 37.0°C. Electrocardiogram showed normal sinus rhythm, and heart rate was 98/min. Laboratory data showed hyperthyroidism, that is, undetectable serum thyroid-stimulating hormone (TSH) (<0.021 *µ*IU/mL; standard range [SR] = 0.541–4.261) associated with elevated levels of both free triiodothyronine (FT3) (8.98 pg/mL; SR = 2.39–4.06) and free thyroxine (FT4) (3.45 ng/dL; SR = 0.71–1.52), with no goiter, no cervical pain, and no thyroidal ophthalmopathy on examination (Figure 1). He was a smoker and had neither a personal nor a family history of thyroid disease. It was necessary to differentiate whether the diarrhea was caused by hyperthyroidism or by another factor. To suppress the excessive secretion of thyroid hormone, we prescribed potassium iodide (100 mg/day). Thyroid-stimulating hormone receptor antibody (TRAb) was positive (4.0 IU/L; SR = <2.0). Thyroglobulin antibody (TgAb) and thyroid peroxidase antibody (TPOAb) were both negative (26 IU/mL; SR = ≤28, <9 IU/mL; SR = ≤15.9, respectively). The thyroglobulin (Tg) level was elevated (276 ng/mL; SR = 0–33.7). Thyroid-stimulating antibody (TSAb) was positive later (211%; SR = ≤120). Thyroid ultrasonography performed the day after iodine treatment showed an increase in the superior thyroid artery flow velocity (Right 50.1 cm/s, Left 44.0 cm/s; ≥50 cm/s as a criterion for increase) and a mild increase in the intrathyroidal blood flow signal (Figure 2). Before the first pembrolizumab administration, his thyroid function was subclinical hyperthyroidism with negative TRAb (<0.5 IU/L). From these results, he was diagnosed as GD which had developed during pembrolizumab administration.

We also suggested that diarrhea had been caused by colitis, and thus we performed colonoscopy. Colonoscopy showed active colitis with granular mucosa, orange peel appearance, and loss of visible vascular pattern. Biopsy samples from terminal ileum, cecum, ascending colon, transverse colon, sigmoid colon, and rectum were active ileitis, typhlitis, and colitis with mild crypt atrophy, cryptitis, and crypt abscess. Stool culture, *Clostridium difficile* toxin, and glutamate dehydrogenase toxin were all negative. Therefore, he was diagnosed as having active colitis with diarrhea as Common Terminology Criteria for Adverse Events Grade 3. His diarrhea had not improved despite the reduction of thyroid hormone with potassium iodide treatment for 10 days. We decided that the diarrhea had been caused by immune-mediated colitis due to pembrolizumab treatment, and not by hyperthyroidism. Subsequently, his diarrhea was rapidly improved by prednisolone 60 mg (1 mg/kg/day).

On the other hand, his thyroid hormone was normalized with undetectable serum TSH for 2 weeks by iodine treatment, and thiamazole 10 mg/day was given in place of potassium iodide. TRAb was not detected after 15 weeks of thiamazole treatment. We were able to precisely document the development of GD induced by pembrolizumab, because his thyroid function was checked regularly. Although pembrolizumab administration was discontinued because of severe diarrhea, there has been no further progression of cancer to date.

## 3. Discussion

The incidences of thyrotoxicosis and hypothyroidism treated with the anti-PD-1 antibody pembrolizumab have been reported by de Filette et al. [4] to be 12.1% and 15.2%, respectively. According to their report, TRAb was tested in five patients and it was found to be elevated in only one patient at the time of thyrotoxicosis. In the one case of positive TRAb, thyrotoxicosis swiftly evolved into hypothyroidism without antithyroid therapy. That patient might have had GD rapidly shifting to hypothyroidism due to a switch in her antibody subpopulation.

Iadarola et al. recently reported a case of nivolumab-induced thyroid dysfunction, GD-like hyperthyroidism [5]. Their 66-year-old male patient, who was administered nivolumab, developed hyperthyroidism (FT4 was in the upper-normal range) with negative TRAb. Thyroid ultrasound showed a multinodular goiter with a normo-echoic pattern of the parenchyma and a normal pattern of vascularization. A 99 mTc scintigraphy revealed a diffuse thyroid uptake of the radionuclide suggesting GD-like hyperthyroidism, and methimazole therapy was started. TRAb test remained persistently negative. That case indicated that GD-like hyperthyroidism can also occur in nivolumab-treated patients, even in the absence of circulating TRAb. The role of thyroid autoantibodies in the pathogenesis of PD-1 inhibitor-induced thyroid dysfunction was debated, but that of TRAb was not discussed and remained unclear.

We herein reported a case in which pembrolizumab-induced hyperthyroidism (FT4 was elevated) with positively converted TRAb. We diagnosed GD considering positive TSAb and the results of thyroid ultrasonography, although thyroid scintigraphy was not evaluated because of iodine treatment. We thought this case was typical GD diagnosed based on TRAb test and thyroid ultrasound data compared to the atypical GD presented in the previous report [5]. In the present case, pembrolizumab-induced GD was improved by potassium iodide and thiamazole and TRAb was converted to negative 15 weeks after the beginning of thiamazole treatment. Concomitantly, immune-related active colitis was also rapidly improved by prednisolone. Therefore, the use of prednisolone may further complicate the course of negatively converted TRAb.

GD is caused by failure of self-tolerance to TSH receptor. Both T cells and B cells are necessary for the development of GD. Large scale genetic analyses have identified several genes including CTLA4 conferring susceptibility of GD. There are only a few reports of GD induced by anti-CTLA4 antibodies [2, 3]. According to Azmat et al., it is plausible to hypothesize that blocking CTLA4 results in the development of activating antibodies against the TSH receptor thereby causing GD in susceptible individuals [2]. Several investigators have also suggested that this relation between CTLA4 and GD are associated with CTLA4 gene polymorphism, which is also true in Japanese [6]. However, the mechanism of GD induced by anti-CTLA4 antibodies is unclear.

In contrast, PD-1 is expressed on T cells and B cells, and PD-L1 is expressed on activated T cells, B cells, cancer cells and antigen-presenting cells. PD-1 prevents T cell proliferation, activation and cytokine release. PD-1 can inhibit B cell receptor signaling and antigen-stimulated B cell activation [7]. In addition, PD-1 is expressed on activated CD4^+^CD25^+^ regulatory T cells (Tregs) in humans [8], and the blockade of PD-1 by antagonistic anti-PD-1 antibodies inhibits the production of IL-10 from regulatory T cells [8] and thereby, enhances lymphocyte proliferation. This suggests that PD-1 may be critical in determining the activation threshold and/or suppressive activity of Tregs. In this way, the PD-1 pathway plays an important role in the down regulation of immune responses and in the prevention of autoimmune diseases. Interestingly however, PD-1 has not appeared on the stage of GD. Reports on the association between PD-1 and GD are few. We introduced two reports which investigated GD and gene polymorphisms of PD-1 [9] and PD-L1 [10]. Newby et al. documented that small effects within PD-1 may contribute towards the development of GD in the UK [9]. Hayashi et al. showed that PD-L1 gene might be one of the candidate gene polymorphisms for GD [10]. It is unclear how the polymorphism affects the development of GD in these studies.

Regarding other drug-induced GD, there was a report stating that the anti-CD52 monoclonal antibody, alemtuzumab, can induce GD in patients with multiple sclerosis [11]; 9 of 27 patients developed GD from 3–31 months after alemtuzumab treatment. The B cell and CD8 lymphocyte counts rose progressively at 15–18 months after alemtuzumab. The development of GD was associated with a quicker recovery of CD8 T cells and a low production of memory CD4 cells. The unique situation has been identified in which biochemical hyperthyroidism can be detected several months before symptoms develop, allowing access to the earliest immunological events in the pathogenesis of GD. Based on this point of view, it may be considered that anti-PD-1 antibody may induce GD by activation of the autoimmune system associated with the development of GD via PD-1/PD-L1 pathway inhibition. However, as the mechanistic profile of anti-PD-1 antibody-induced GD remains unclear, further study and the accumulation of case reports regarding ICI-induced GD is necessary.

In conclusion, this case report of GD with positive TRAb induced by the anti-PD-1 antibody pembrolizumab may contribute to the understanding of the mechanism behind the association of the development of GD and autoimmune activation via PD-1.
